# Report of Surgeon-General W. K. Van Reypen, U. S. Navy

**Published:** 1898-11

**Authors:** 


### Selection.

### REPORT OF SURGEON-GENERAL W. K. VAN REYPEN, U. S. NAVY.

[*Abstract.*]

. . . . . . . .

IN THIS report precedence is naturally given to the operations of the bureau during the war with Spain. From the time of the blowing up of the Maine in the harbor of Havana, on February 15, 1898, preparations were made by the bureau providing for any possible contingency. The naval hospitals were equipped to their full capacity; plans were prepared for building pavilion wards on the hospital grounds to give accommodation to any number of sick or wounded that the bureau might be called upon to care for. The director of the naval laboratory prepared to furnish medical and surgical supplies in any quantity, at any place, and immediately. No additional expense was incurred until war seemed imminent; then the vessels that were likely to be engaged were supplied with a full outfit of supplies for war. In anticipation of a large number of additional vessels being taken for service, medical and surgical outfits of a kind suitable for the various classes of vessels were bought, assembled and boxed, ready to be shipped anywhere as soon as called for. There has not been an instance during the war of any vessel having to wait for her medical stores.

It was known that a corps of volunteer medical officers would be a necessity, and before war was declared or any law passed authorising their employment medical boards of examination were established in Boston, New York, Philadelphia, Washington, Norfolk and Mare Island (Cal.) to examine applicants for appointment, such appointment being contingent upon their services being required. As the result of their examinations, a waiting list of well-educated medical men was ready, from which appointments were made as soon as their services were required after the declaration of war. Over 2,000 applications were received, but only a small proportion examined. Out of this number thirty-seven were appointed assistant surgeons. They have rendered efficient service, and have been a credit to the navy. Some have had unusual and trying experiences, but they have accommodated themselves to their environments and have justified their appointments.

One of their number, Assistant Surgeon John Blair Gibbs, was killed in the action at Guantanamo while serving with the marine battalion. He was the only medical officer killed during the war.

### FITTING OUT THE SOLACE.

The question of proper care and transportation of sick or wounded at sea had long been a subject of consideration by the bureau. The coming of war gave it an opportunity to demonstrate the wisdom of its propositions and the efficiency of its methods. By direction of the President, and by the authority of the Secretary of the Navy, the steamer Creole, of the Cromwell line, between New York and New Orleans, was purchased and designated as an ambulance ship. The vessel was sent to the yard of the Newport News Shipbuilding & Dry Dock Company and there fitted out on the plans of the bureau. The work was done under the bureau of construction and repair and under the immediate superintendence of naval constructor J. J. Woodward, to whom the bureau is under lasting obligations for his advice, assistance and his energy in satisfactorily completing the work. The merchant ship Creole became the ambulance ship Solace in sixteen days, fitted with a well-lighted operating room, in which were all the appliances for modern antiseptic surgery, a steam disinfecting apparatus, an ice machine, a steam laundry plant, cold storage rooms, and a elevator for taking patients from the operating room and upper deck to the wards below.

The Solace is fitted out under the requirements of the Geneva convention, and flies the Geneva cross flag. She is a pioneer in her work and indicates a step in advance that it well became the United States to take. Her fitting out was easy of accomplishment. The chief of every bureau in the department having to do with the vessel gave his cordial support and assistance to the work. They gave the Solace everything she needed. The vessel has been fortunate in her personnel. Commander Dunlap is an ideal commander and the medical officers of the vessel, Surgeon Streets and Passed Assistant Surgeons Stokes, Smith and Bogert, have shown themselves thoroughly competent and efficient in caring for the many sick or wounded who have been under their charge. Three hospital stewards, one of whom was a skilled embalmer; eight trained nurses, a cook, four mess men and two laundrymen were especially designated for service in the medical department.

DESCRIPTION OF THE VESSEL.

The Solace is built of steel, 3,801 tons, 375 feet long, 44 feet beam, draws 21 feet, and has a continuous speed of 16 knots. She can comfortably accommodate 200 patients, either in birth, swinging cots or staterooms. The hurricane deck aft is inclosed with canvas, for use as a contagious ward, if required. She carries 37,000 gallons of fresh water in tanks and 800 tons in her double bottom. Distillers and evaporators keep up the supply. As soon as the Solace received her stores she sailed for the blockading squadron, and arrived in time to take on board the wounded at the bombardment of San Juan. She then collected the sick or wounded from the other vessels of the squadron and sailed for New York, where on June 5th fifty-seven patients were landed at the naval hospital. On June 8th she sailed for Guantanamo, and was present to take on board the wounded marines in their fight with the Spanish troops.

As soon as the Spanish fleet was destroyed in the battle of July 3d, she took on board the wounded from the Brooklyn and all the Spanish wounded and gave them the care and attention that have never before been given to the wounded of friend or foe in any naval combat and that could only be given by an ambulance ship. As it was the policy of the department to bring all the sick or wounded from Southern waters to Northern naval hospitals, as soon as practicable, so that they might have a better chance for recovery, and as there was still space left on the Solace for wounded men, she went to Siboney and took on board forty-four army wounded and sailed for Hampton Roads on July 12th. On July 16th she landed forty-four army wounded at Fortress Monroe, and fifty-five navy sick or wounded, and forty-eight Spanish wounded at the naval hospital Norfolk. She then went to New York for coal, stores and an additional ice plant, and sailed on August 2d for Key West, where she took on the sick from the hospitals and vessels in port, and then visited all the vessels on the blockade around Cuba, taking off their sick and wounded and leaving stores. After receiving at Guantanamo the sick brought by the Gloucester from the vessels around Porto Rico, she sailed for Boston, and on August 29th landed seventy-four sick from the navy and two sick soldiers at the Chelsea Naval Hospital. She then coaled and went to New York for repairs and stores and sailed on September 22d for Guantanamo with orders to deliver stores and supplies to all vessels in Cuban or Porto Rican waters, take on board their sick, and then return to New York, bringing in addition as many sick and wounded of the army as the vessel could accommodate.

### GENEROUS SUPPLIES FOR THE SICK.

On every trip of the Solace she has gone loaded with medical stores and supplies, and also with delicacies and comforts, which have been supplied in abundance for the sick or wounded by generous and patriotic individuals and societies from every part of the United States. Among the contributions to the Solace were a carbonator and deck awning from the Rhode Island Sanitary and Relief Association, an *x*-ray apparatus from the National Society of Colonial Dames, and conveyance boxes for sterilised dressings from the Elizabeth (N. J.) members of the National Society of Colonial Dames.

In this war woman has done her perfect work and the medical department of the Navy is profoundly grateful for the money contributed and supplies furnished for the aid and comfort of the sick or wounded of the navy. Patriotic women have ably supplemented the efforts of the government, and their assistance has been thoroughly appreciated.

The contributions soon became so numerous that it was necessary to have a medical officer detailed to receive them. Medical Director Bloodgood was assigned to the duty, and he has received and distributed the stores and attended to the voluminous correspondence with the same business ability he manifested when on the active list.

As soon as war was declared the daughter of the Secretary of the Navy and three of her associates at the Johns Hopkins Medical School volunteered their services as nurses, and were assigned to duty at the Naval Hospital, Brooklyn, N. Y. Six women nurses from the registered list of the Daughters of the American Revolution and five Sisters of Charity at Norfolk also volunteered, and were assigned to duty at the Naval Hospital, Norfolk, Va. All of these women have done their work thoroughly and conscientiously; they have the thanks of the navy and the gratitude of their patients.

The medical officers of the naval reserves, who were transferred to the service with the reserves from their states, rendered efficient service and willingly responded to every call made upon them.

The bureau is under obligations to the Surgeon-general of the army and to the Supervising Surgeon-general of the Marine Hospital Service for caring for the sick and wounded of the navy in the hospitals under their charge at Key West.

### PRISONERS QUICKLY PROVIDED FOR.

When the department decided to remove the prisoners from the destroyed Spanish fleet at Santiago to Portsmouth, N. H., immediate preparation was necessary to care for the sick. Two pavilions were built from plans already prepared, adjoining the Naval Hospital, at Portsmouth. Telegraphic orders were issued for bedsteads, mattresses, bedding, stores and supplies. Additional medical officers and nurses were sent, and when the vessels arrived with the prisoners the well men found comfortable barracks and the sick comfortable hospitals, to which they were immediately transferred. They were lodged, fed and clothed as though they were expected guests. The navy has reason to feel proud of this five days' work. One hundred equipped cots and six trained nurses were generously supplied by the Red Cross after the hospital was established.

On June 17th the President approved an act of Congress organising a hospital corps of the navy. The passage of this act is the culmination of the efforts of the bureau for many years. It will give the service a trained corps of men who will now have some reason for remaining in service, having a hope of promotion and advancement as the result of faithful service, sobriety and attention to duty. Its good results are already manifest. Changes are being made as rapidly as practicable, and nearly all of the hospitals are now supplied with trained nurses, and in many of them are apprentices undergoing instruction. The examination for admission is rigid, and there will be more admissions to the corps when the end of the war releases from service many of the trained nurses now employed in other departments.

I cannot close this portion of the bureau's report without bearing testimony to the efficiency, skill and devotion to the duty of the personnel of the medical department. Not a word but of praise has the bureau heard of any of them—regulars or volunteers. When war was imminent they vied one with another in their efforts to get on fighting ships. Some have had greater opportunities than others, but all have done well the work assigned them. Surgeon Edgar saw his associate, Assistant Surgeon Gibbs, shot by his side in the Spanish attack, and he continued his work alone, doing it thoroughly and well, as it was known he would.

The medical officers of the vessels in the fight at Manila and in the battle of July 3d, shared the dangers of their comrades, and should participate in the praise accorded to them.

ANTISEPTIC SURGERY AT SEA.

The medical officers of the Solace have the honor of inaugurating the first complete system of antiseptic surgery at sea. They have adapted means to ends, have improvised apparatus, have been fertile in expedients and have the satisfaction of having demonstrated that, with skill and intelligence, the percentage of mortality among the patients on a well-equipped ambulance ship will be no greater than in the hospitals on shore.

Medical Inspector Persons found himself suddenly confronted with 226 Spanish sick or wounded prisoners in a hastily established hospital. He was equal to the emergency, and he and his associates were complimented by Admiral Cervera when he visited the camp.

The medical officers of the other hospitals have had sudden large accessions of patients. They were always ready and always cared for them well.

The director of the laboratory (our receiving and distributing depot of supplies) applied his well-known energy to the work, and never failed to have supplies ready whenever and wherever required. Those whose services has not been so conspicuous have done their duty in the stations assigned them, and have contributed their share toward the efficiency of the medical department of the navy.

STAMPING OUT YELLOW FEVER.

. . . . . . . .

Upon the commencement of hostilities between this country and Spain, the navy department ordered a flag officer in command of the naval station at Key West, Fla., and a short time afterward designated it as a naval base for all vessels acting in southern waters.

In order to provide for all possible contingencies and to meet the demands incident to a state of war that might be made upon it as a result of assembling in this harbor so large a number of war vessels, the department landed at Key West a quantity of naval supplies, including ordnance stores, equipment, materials, provisions, etc.

For the preservation of this property (valued at hundreds of thousands of dollars) it was deemed essential that suitable provision should be made for its protection. Early in June, therefore, fifty-four officers and men of the marine corps were transferred to Key West and located in a building well adapted for temporary quarters, and which had formerly been used as a cigar factory.

For several years previous to the present summer, yellow fever, with the exception of an occasional sporadic case, had not made its appearance in Key West, but the location of the island, well within the limit of the yellow-fever zone, and the sudden concentration at this point of a large number of unacclimated persons belonging to the army and navy, rendered it highly probable that, unless extra precautions were taken for the preservation of health, an outbreak of yellow fever could only be postponed for a short time.

The general unsatisfactory sanitary condition of Key West prevailing at this time also, was such as to cause some uneasiness on the part of the department, as it was but reasonable to infer that the exemption of the island from yellow fever for the last few years might be attributed to accident, rather than to any special attention on the part of the officials to the enforcement of hygienic measures.

### THE FIRST CASES AT KEY WEST.

Although the duties devolving upon the marines were arduous and exacting, the health of the guard remained good until August 13th, when the first suspicious case of illness among them made its appearance. On the morning of August 14th another marine was taken ill, and on the afternoon of August 15th three more cases were reported. The symptoms in all of the above cases were expremely suspicious, but before making a positive statement Assistant Surgeon Marcour decided to avail himself of the opinions of several yellow-fever experts as to the diagnosis in the above cases. After a consultation between the local health officers of Key West and representatives from the medical corps of the army, navy and marine hospital service, a definite conclusion was reached as to the nature of the disease, and on August 16th the bureau was informed officially of the existence of yellow fever and that a rigid quarantine had been established at Key West by the national and local health authorities.

Upon the receipt of this information the commandant in charge of the United States naval base was instructed by the department to send at once all naval vessels in the harbor of Key West to Hampton Roads (including the officers and men on temporary shore duty, with the exception of the marine guard), and to transfer to some suitable vessel for passage north the sick and wounded of the navy who could be removed without danger from the army general and marine hospitals. Orders were also issued that no naval supplies stored in the several buildings of Key West should be removed, as it was not deemed safe to transfer them at this time for fear of spreading the infection. The commandant was further directed to have the sick marines isolated within the barracks then occupied by them, to place all suspects or suspicious cases in an adjoining house rented for the purpose, and to remove the well marines to the detention camp, which was situated on the south beach at a distance of about three miles from the barracks. Every provision was made for the care and treatment of the sick, and every precaution adopted for the protection and preservation of the health of the marines in the detention camp.

LOCALISING THE DISEASE.

Assistant Surgeon R. F. Marcour (a yellow-fever immune) had been selected by the bureau to accompany the marines when ordered on this duty, in anticipation of a possible outbreak of yellow fever. Eight immune nurses, one immune cook and one immune watchman were also employed. On August 18th the number of cases under treatment had increased to ten, and as there appeared to be at that time every probability that the disease would develop in an epidemic form, the bureau decided to detail an additional medical officer for this duty.

Surgeon John W. Ross (a yellow fever immune) was temporarily detached from the navy yard, Pensacola, Fla., and on August 23d arrived at Key West and assumed charge of all yellow-fever cases.

The prompt measures, however, adopted by the medical officers in the immediate and complete isolation of the sick, the thorough disinfection of the quarters occupied by them and the removal of the well marines to a place beyond the danger point of infection, resulted in localising the disease, and on September 12th Surgeon Ross reported the appearance of the last case—that of Commander Forsyth—making a total number of patients under treatment from the beginning of the epidemic on August 13th until its close on September 12th of fourteen cases. The disease prevailed in a mild form, and up to this time no deaths have occurred.

Upon the recommendation of Surgeon Ross, and with the approval of the bureau, the department ordered the transfer north of the marines, and on September 8th the guard left Key West on the steamship Colorado, and arrived in New York on September 14th.

The Colorado was subjected to a thorough process of disinfection at the quarantine station, New York, and the bedding, mattresses, and the like, of the marines were sent to the Navy Hospital, New York, where they were thoroughly disinfected before being placed in the barracks.

In conclusion, Surgeon-general Van Reypen gives an insight into the care which is exercised in securing good material for the medical corps of the navy, which goes far to explain the efficiency of this arm of the service. It is shown that in the last fiscal year 829 applications for information concerning the appointments of assistant surgeons in the navy were received, and that 248 permits were issued for doctors to appear for examination.

Of the above sixty-five candidates appeared before the examining boards, of which seventeen were rejected physically, nineteen rejected professionally, twelve withdrew from further examination and seventeen were found physically and professionally qualified for admission as assistant surgeons in the medical corps of the navy.

. . . . . . . .

Upon the recommendation of the bureau and with the approval of the department, Congress in the last session very wisely extended the age limit from twenty-six to thirty years for entrance into the medical corps of the navy.

This legislation has been attended with most gratifying results, and for the first time in thirty-five years the number of officers in the medical corps of the navy has reached the limit established by law.

